# Macrophage Autophagy and Oxidative Stress: An Ultrastructural and Immunoelectron Microscopical Study

**DOI:** 10.1155/2011/282739

**Published:** 2011-09-13

**Authors:** Ida Perrotta, Valentina Carito, Emilio Russo, Sandro Tripepi, Saveria Aquila, Giuseppe Donato

**Affiliations:** ^1^Department of Ecology, Faculty of Mathematical, Physical and Natural Sciences (SMFN), University of Calabria, 87036 Cosenza, Italy; ^2^Department of Pharmaco-Biology, Faculty of Pharmacy, University of Calabria, 87036 Cosenza, Italy; ^3^Department of Experimental and Clinical Medicine, School of Medicine, University Magna Graecia, 88100 Catanzaro, Italy; ^4^Department of Pathology, School of Medicine, University Magna Graecia, 88100 Catanzaro, Italy

## Abstract

The word autophagy broadly refers to the cellular catabolic processes that lead to the removal of damaged cytosolic proteins or cell organelles through lysosomes. Although autophagy is often observed during programmed cell death, it may also serve as a cell survival mechanism. Accumulation of reactive oxygen species within tissues and cells induces various defense mechanisms or programmed cell death. It has been shown that, besides inducing apoptosis, oxidative stress can also induce autophagy. To date, however, the regulation of autophagy in response to oxidative stress remains largely elusive and poorly understood. Therefore, the present study was designed to examine the ratio between oxidative stress and autophagy in macrophages after oxidant exposure (AAPH) and to investigate the ultrastructural localization of beclin-1, a protein essential for autophagy, under basal and stressful conditions. Our data provide evidence that oxidative stress induces autophagy in macrophages. We demonstrate, for the first time by immunoelectron microscopy, the subcellular localization of beclin-1 in autophagic cells.

## 1. Introduction

The term autophagy (from the Greek “auto” for “self” and “phagein” meaning “to eat”), was first used in the 1960s by Christian de Duve and refers to a series of intracellular processes that lead to the removal of cytosolic proteins or entire cell organelles through lysosomes [1]. In mammalian cells, the three main autophagic pathways, macroautophagy, microautophagy, and chaperone-mediated autophagy (CMA) are frequently interconnected and share some common components. Macroautophagy (hereafter simply referred to as autophagy) has been originally described as a cellular adaptation to starvation involving the sequestration of cytoplasmic proteins and organelles into a closed, double membrane structure, called the autophagosome that consequently fuses with lysosomes to form an autolysosome, where the final hydrolytic degradation of the engulfed cytoplasmic material takes place [2–4]. Although autophagy appears to operate broadly as a cell survival mechanism, a dysregulation of autophagic flux may also be associated with cellular toxicity and may potentially contribute to the development of pathological conditions through excessive depletion of essential cytoplasmic components [5]. The regulation of autophagy has been extensively studied in the past few years especially under nutrient deprivation conditions; however, most mammalian autophagy proteins are still not characterized at all and very little is known about the autophagic response to stress and/or pathological damage [6–8]. Beclin-1, a mammalian orthologue of the yeast Apg6/Vps30 gene, is the first identified mammalian gene with a role in mediating autophagy and appears to act as a nexus point between autophagy, endosomal, and perhaps also cell death pathways [9, 10]. Although the molecular properties of beclin-1 have been characterized, its subcellular localization has not been unequivocally determined [11, 12]. Reactive oxygen species (ROS) have a complex relationship with autophagy and beclin-1 expression [13–15]. ROS (superoxide, hydrogen peroxide, hydroxyl radical, and other related compounds) are produced continuously in cells as consequence of both enzymatic and nonenzymatic reactions and regulate a diverse array of physiological functions [16]. However, when under certain conditions the production of ROS becomes excessive, the resultant oxidative stress can negatively affect biomolecules (such as proteins or DNA) and can alter the functional and structural integrity of biological tissues [17]. ROS not only cause a direct damage of cell constituents, but also may serve as important stimuli of autophagy becoming involved in several pathways that regulate both cell survival and death [18]. Even if different methodological approaches have been adopted to detect autophagy, electron microscopy remains currently the most accurate technique that allows the visualization of the morphological events and molecular mechanisms of autophagy and provides a faithful demonstration of the ongoing of autophagic activity within cells. In the present study we show, for the first time by immunoelectron microscopy, the subcellular localization of beclin-1 in autophagic cells and further evidence that autophagy is a cellular mechanism closely related to oxidative stress.

## 2. Materials and Methods

### 2.1. Macrophage Isolation and Culture Conditions

Peripheral blood mononuclear cells (PBMCs) were obtained from male healthy donors (25–30 years old). Buffy coat preparations from healthy donors were diluted 1 : 1 with phosphate-buffered saline solution (PBS) and centrifuged over Ficoll-Paque solution (GE Health Care, Milan, Italy). Monocytes were separated from lymphocytes by adherence as described previously with minor modifications [19]. PBMCs were cultured at 37°C in humidified atmosphere enriched with 5% CO_2_ in RPMI 1640 supplemented with 1% (v/v) of penicillin/streptomycin and 10% (v/v) of fetal bovine serum heat-inactivated and endotoxin free. After 7 days of culture, nonadherent cells were removed by repeated washings with warm medium. Macrophages obtained with this method resulted in >95% of purity by cytofluorimetric analysis. To monitor the ability of oxidative stress to induce autophagy, macrophages were incubated with free radical generator 2,2-azobis (2-amidinopro-pane) dihydrochloride (AAPH, 5 mmol/L) in Dulbecco's modified Eagle's medium without serum at 37°C in the dark for 5 h.

After this incubation period, cells (untreated controls and AAPH-treated macrophages) were detached with trypsin treatment, concentrated by gentle centrifugation, and adequately processed for electron microscopy.

### 2.2. Transmission Electron Microscopy

For routine transmission electron microscopy, cell pellets were fixed in 3% glutaraldehyde solution prepared in 0.1 M phosphate buffer at 4°C, postfixed in osmium tetroxide (3%) for 2 hours, dehydrated in graded acetone, and embedded in Araldite (Fluka, Buchs, Switzerland). Ultrathin sections were prepared using a diamond knife, collected on copper grids (G 300 Cu), contrasted using both lead citrate and uranyl acetate, and then examined with a “Zeiss EM 900” electron microscope.

### 2.3. Immunoelectron Microscopy

Control and AAPH-treated macrophages were fixed in 4% paraformaldehyde + 1% glutaraldehyde solution in 0.1 M phosphate buffer (pH. 7.4) overnight at 4°C. Cells were dehydrated at room temperature through graded ethanol solutions, then infiltrated in an intermediate solution of LR white acrylic resin (London Resin, Berkshire, UK) and 70% ethanol (2 : 1) for 1 h, and embedded in fresh LR white resin overnight at 4°C. The resin was polymerized in gelatin capsules for 24 h at 50°C. Ultrathin sections were prepared using a diamond knife and collected on Formvar carbon-coated nickel grids. For indirect immunolabeling, grids with sections were floated on drops of 1% bovine serum albumin in PBS containing 0.02 M glycine and normal goat serum at room temperature for 30 min to reduce nonspecific binding. Sections were successively incubated with a rabbit polyclonal antibody to beclin-1 (1 : 10) (Santa Cruz Biotechnology, Heidelberg, Germany) in PBS + 0.1% BSA at 4°C overnight. After the grids had been washed rigorously several times with drops of PBS + 0.1% BSA, they were then incubated with 10-nm *γ*-globulin goat antirabbit—gold particle complex at a dilution of 1 : 10 for 1 h at room temperature. Control staining to demonstrate immunohistochemical specificity included replacement of primary antibody by nonimmune goat serum at 4°C overnight. After immunolabeling, the sections were washed with PBS + 0.1% BSA, further washed with distilled water, dried, and then stained with uranyl acetate. All sections were examined with a “Zeiss EM 900” electron microscope.

## 3. Results

### 3.1. Ultrastructural Analysis of Monocyte-Derived Human Macrophages Following Oxidative Stress

The ultrastructure of cultured blood monocyte-derived human macrophages was investigated and correlated under the effect of AAPH-induced oxidative stress (dose = 5 mmol/L).

**Figure 1:**
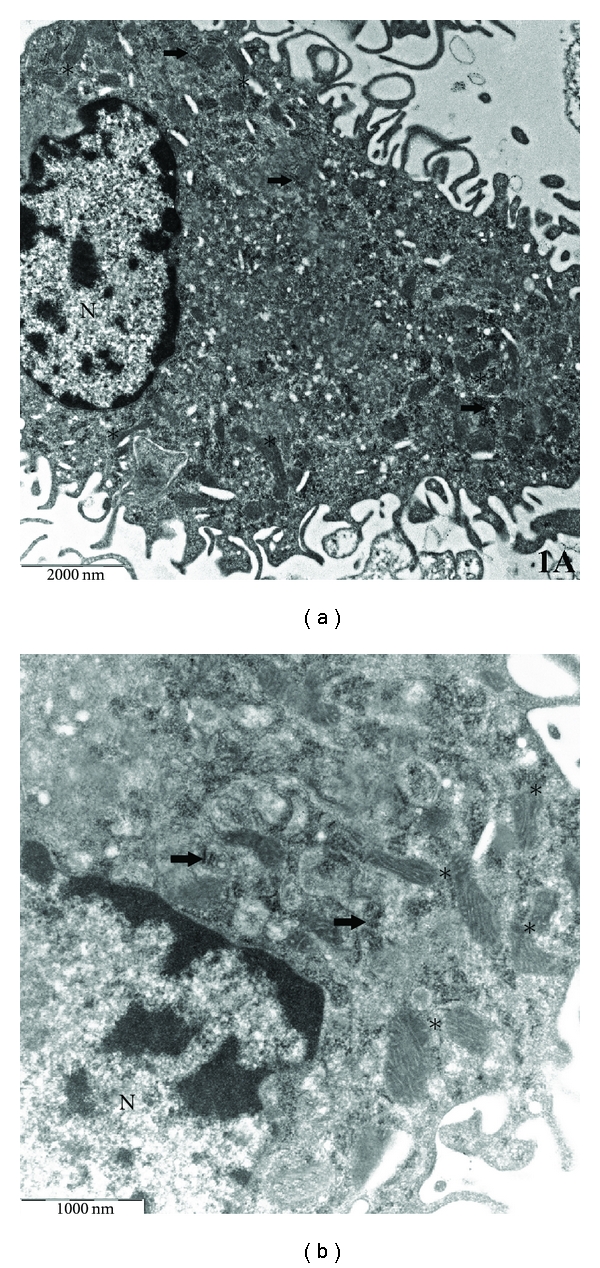
Ultrastructure of a normal, untreated human monocyte-derived macrophage. The cell possesses a finely vacuolated cytoplasm and, usually, numerous pseudopodia. The cytoplasm contains large mitochondria (asterisks) and a variable amount of free ribosomes and rough endoplasmic reticulum rER (arrows). ((a) ×10000; (b) ×25000). N: nucleus.

Control macrophages were irregular in shape with a finely vacuolated cytoplasm and numerous folds, processes and pseudopodia pushed out in all directions. Cells showed prominent profiles of rough endoplasmic reticulum and Golgi apparatus. Mitochondria were characteristically abundant and easily distinguishable. They were either elongated or rounded with simply tubular cristae. Vacuolar and granular structures, probably lysosomal in nature, were only poor developed. The nucleus occupied an eccentric position within the cell and appeared rounded or ovoid in shape with a thin rim of condensed chromatin (Figures 1(a) and 1(b)).

**Figure 2:**
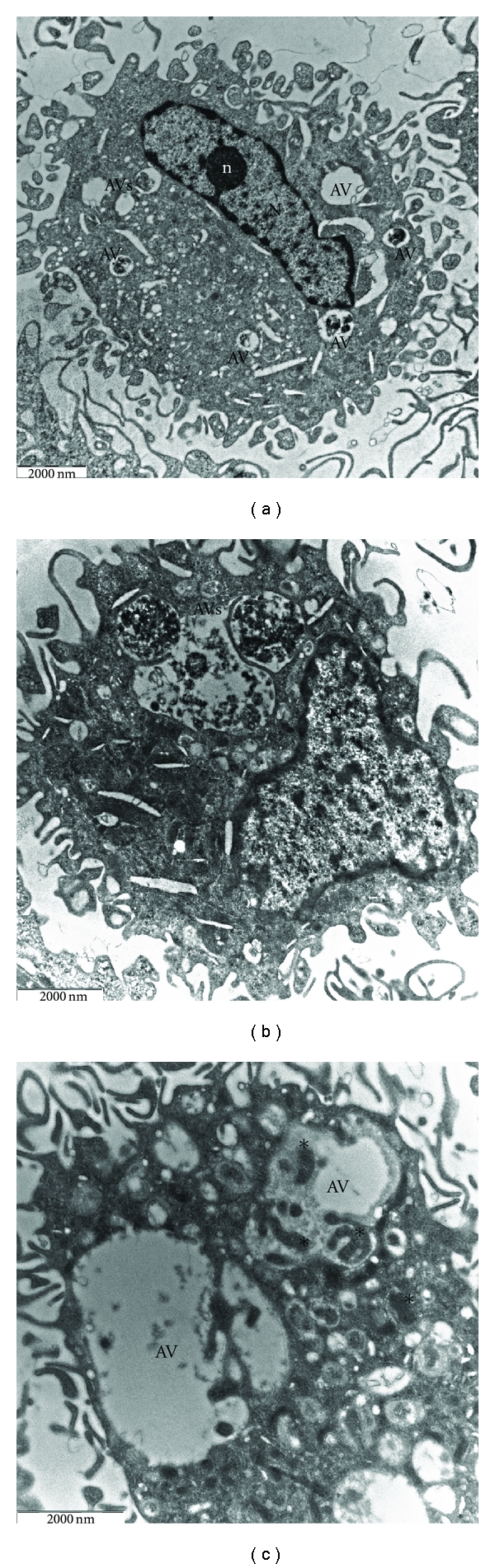
Ultrastructure of AAPH-treated macrophages. Nuclei appear intact with well-preserved nucleoli, a distinct nuclear envelope, and normal chromatin structure ((a) ×8000). Cells show the presence of many autophagic vacuoles of different sizes that enclose cytoplasmic components ((b) ×10000; (c) ×12500). N: nucleus; n: nucleolus; AV: autophagic vesicle; asterisks indicate mitochondria.

In AAPH-treated groups, the cell size remained almost unchanged, nuclei displayed a smooth surface and a normal ellipsoidal shape with well-preserved nucleoplasmic and nucleolar components. The nuclear envelope also remained intact (Figure 2(a)).

**Figure 3:**
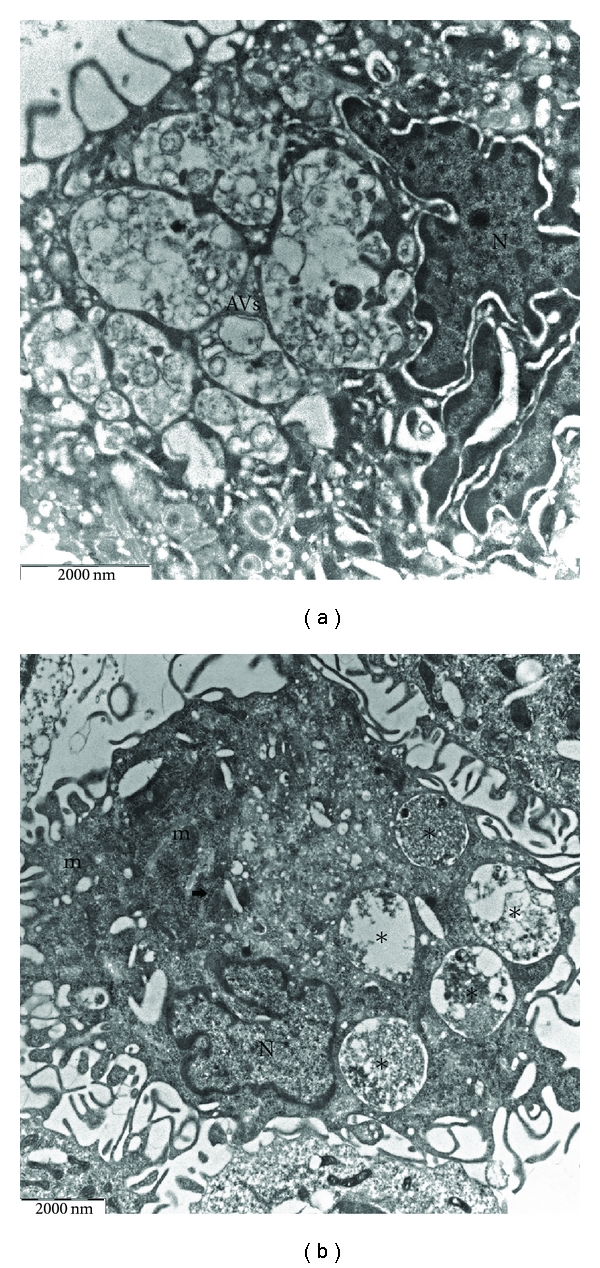
In the AAPH-treated groups, some cells appear to consist almost entirely of autophagic vesicles (asterisks), while others contain only a few vacuoles ((a) ×12500; (b) ×8000). N: nucleus; m: mitochondria; arrow indicates RER.

The most striking feature evoked by oxidative stress was the increase in the number of lysosomes accompanied by the presence of multiple, cytoplasmic membrane-bound vesicles containing portions of the cytosol and membrane-like structures, probably from sequestered cytoplasmic organelles (Figures 2(b) and 2(c)). These structures were diagnostic of autophagy. In some cases, the sequestered material appeared ultrastructurally intact, while in other cases it showed loss of distinct morphological features as a sign of ongoing degradation. The alterations described above tended to vary in degree from cell to cell. In some macrophages, the cytoplasm was almost filled with autophagic vacuoles, containing electron-dense elements and partially degraded material; intracellular organelles were also greatly reduced in number (Figure 3(a)). In contrast, other macrophages showed only moderate amounts of autophagic vacuoles, and cell organelles tended to accumulate in normal cytoplasmic areas (Figure 3(b)). Treated cells did not show morphological evidence suggestive of apoptosis, such as nuclear shrinkage, margination and condensation of the chromatin, enlargement of endoplasmic reticulum cisternae, and convolution of the cell with formation of apoptotic bodies.

### 3.2. Analysis of Oxidative Stress-Induced Autophagy Using Immunogold EM

**Figure 4:**
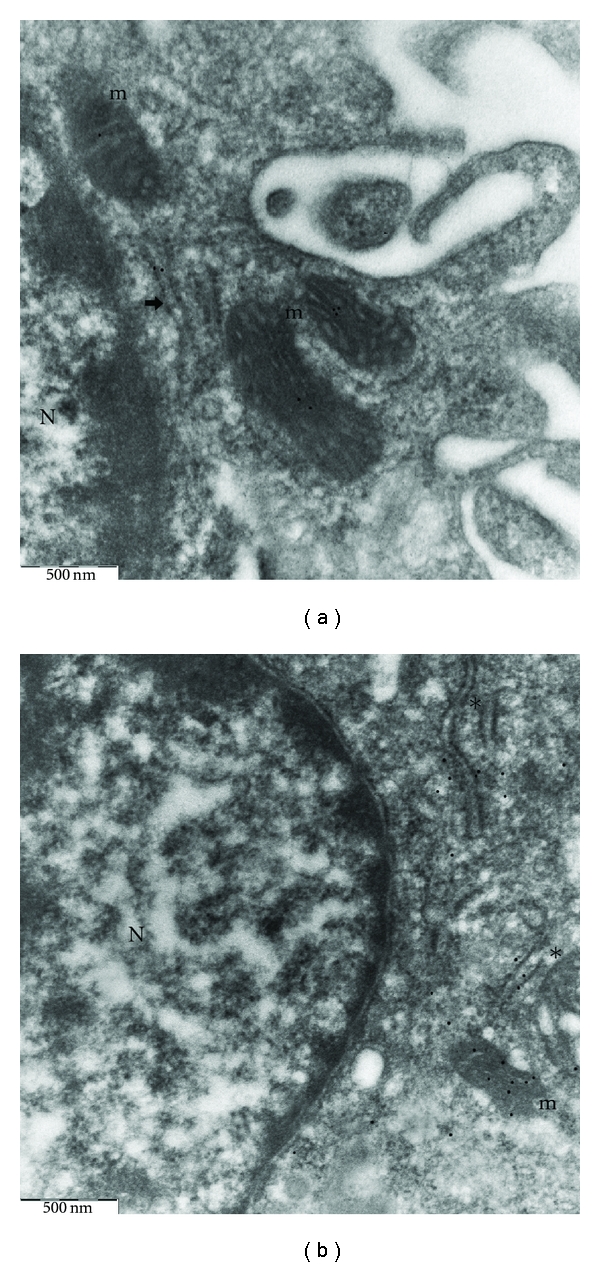
Immunoelectron microscopy of beclin-1 in control macrophages. Gold particles are present within the cytoplasm and organelles, such as rER (asterisks) and mitochondria but not in the nucleus ((a) ×50000; (b) ×40000). N: nucleus; m: mitochondria; arrow indicates RER.

**Figure 5:**
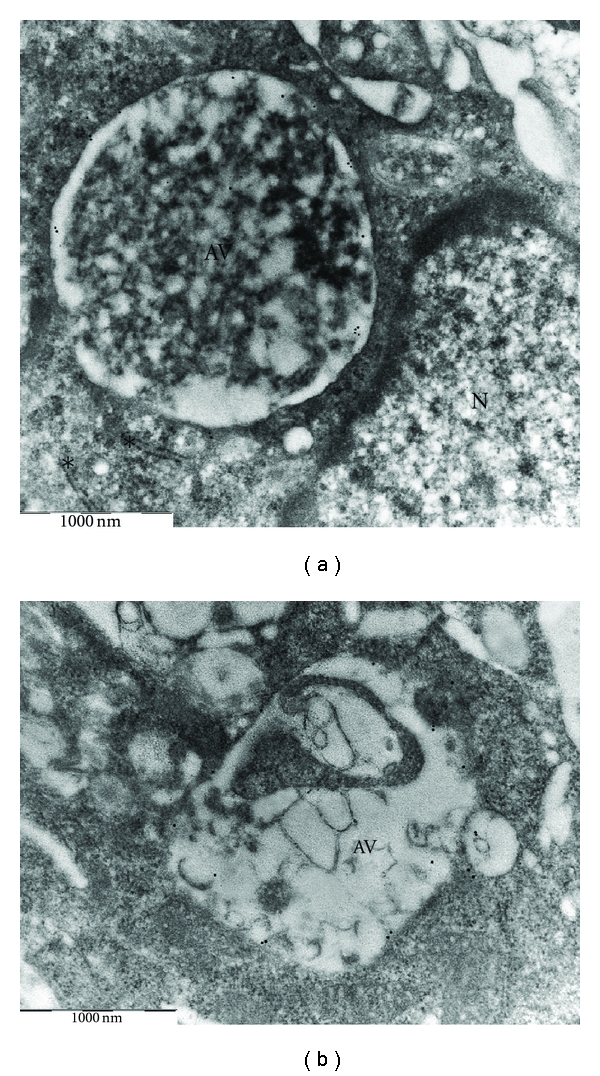
Immunoelectron microscopy of beclin-1 in AAPH-treated macrophages. Gold particles specifically localize to autophagic vesicles. Label occurs predominantly over the enveloping membranes ((a) ×31500; (b) ×31500). N: nucleus; AV: autophagic vesicle; asterisks indicate RER.

Antibody against beclin-1 was used to precisely define the subcellular localization of the protein of interest in both basal conditions and upon oxidative stress. Rabbit pAb to human beclin-1 recognized only the 60 KDa beclin-1 protein of mouse, rat and human origin, and no additional proteins. A distinct cytoplasmic labeling was found in normal, untreated macrophages (Figures 4(a) and 4(b)). Mitochondria and RER were the only significantly labelled elements in the cytoplasm. Beclin-1 immunolabeling was noted on the RER membranes and in the spaces between them. In the mitochondria, gold particles were associated with the outer, as well as the inner, mitochondrial membrane. The cell nucleus was not labelled. To explore whether beclin-1 was involved in the process of cell autophagy under conditions of oxidative stress, cultured macrophages were exposed to 5 mmol/L AAPH. Immunogold analysis revealed translocalization of beclin-1 to the autophagic vesicles (Figures 5(a) and 5(b)). The protein was mainly localized to the membranes of autophagic bodies; however, some gold particles were also found inside the lumen of these structures. The staining was seen in autophagic vesicles containing mitochondria, membrane-like structures, or other degenerated material. Almost no gold markers were present on RER cisternae and in the nucleus. Also the mitochondria were totally free of labelling. Beclin-1 immunoreaction was specific because (i) there was no labeling in the absence of the primary antibody and (ii) the reaction was strictly confined to the cells and only to specific compartments (iii) with no signal on the embedding medium.

## 4. Discussion

Oxidative stress, which is mediated by reactive oxygen and nitrogen species (ROS and RNS, resp.) has been increasingly related to the onset and/or progression of many human disorders ranging from cardiovascular to neurodegenerative and inflammatory diseases [20]. ROS and RNS are known to interact with cellular biomolecules such as membrane lipids, proteins, and nucleic acids, thereby resulting in alteration and/or disruption of cellular functions and membrane integrity and eventually leading to cell death [21, 22]. Accumulation of reactive species has also been proposed to induce autophagy [23, 24]. To date, however, the molecular machinery that promotes autophagy during oxidative stress is still very poorly understood.

Herein, we demonstrate, that human monocyte-derived macrophages (HMDMs) exposed to AAPH undergo an active autophagic activity characterized by the formation of numerous membrane-bounded vesicles analogous to the autophagosomes previously described in various mammalian cells, plants, and yeast system [25–28]. Furthermore, we show that under conditions of oxidative stress induction of autophagy correlates with a redistribution of Beclin-1, an autophagy-related protein, from the cytoplasm to the autophagosomes.

AAPH is a well-known trigger of free radical-mediated oxidative reactions capable of inducing hemolysis, apoptosis, cell death, and enzyme inactivation because of its unique ability to generate peroxyl radicals. Besides the above mentioned effects, addition of AAPH to a cell culture system has been reported to increase intracellular ROS concentration and lipid hydroperoxide levels in cellular membrane resulting in oxidative stress [29].

Cellular oxidative stress has been shown to serve as an important stimulus for autophagy during periods of nutrient starvation, ischemia/reperfusion, hypoxia, and in response to cell stress promoting either cell survival and/or death [18, 30, 31]. Under conditions of oxidative stress, ROS are generated at high enough levels to cause oxidation and damage to DNA, lipids, proteins, and other cell constituents. Previous reports have proposed that increased autophagy may represent a second level of defense, when antioxidant activities are compromised, providing a selective advantage to minimize the effects of oxidative stress and conferring resistance to oxidant insults [32]. In this context, autophagy may significantly promote cell survival by removing damaged cytosolic components, such as ribosomes (ribophagy), peroxisomes (micropexophagy and macropexophagy), endoplasmic reticulum (reticulophagy), parts of the nucleus (piecemeal microautophagy of the nucleus), and even mitochondria (mitophagy) [33].

In addition to a cell-autonomous role for autophagy in promoting survival, accumulating data suggest that autophagy may also regulate programmed cell death [34, 35]. For instance, it has been demonstrated that after LPS treatment, and in the presence of caspase inhibitors, ROS can induce membrane blebbing and autophagic cell death in cultured macrophages through a caspase-independent pathway [36]. It has been also shown that under oxidative stress blockage of caspase, activation may trigger an “autophagy-related vicious circle” which enhances intracellular ROS levels via the selective degradation of catalase leading to a further increase in ROS and finally to autophagic cell death in mouse fibroblasts [37]. More recently, other studies showed that increased oxidative stress results in the induction of endoplasmic reticulum stress, which, in turn, can lead to autophagic cell death through the activation of the JNK/p38 signaling pathway [38].

Although different approaches have been applied to detect autophagy, electron microscopy still remains the “gold standard” method for assessing this phenomenon [39]. Unlike apoptosis, autophagy-mediated cell death pathway occurs whithout caspases activation, and therefore the nucleus appears intact and cellular fragmentation is absent [40]. On the basis of these criteria, we demonstrate by ultrastructural analysis, that under conditions of oxidative stress macrophages enlist autophagy accumulate autophagic vesicles but do not show morphologic hallmarks of apoptosis. Apart from being involved in the innate and adaptative immune response to bacteria, viruses, and parasites [41, 42], macrophages autophagy has been implicated in the pathogenesis of various oxidative stress-related diseases such as Crohn's disease (CD) and atherosclerosis. For instance, it has been demonstrated that defective autophagy modifies the handling of intracellular bacteria in Crohn's granulomas, while in the atherosclerotic plaque autophagy it may play role in the selective clearance of macrophages, thus, representing a potential mechanism of lesion stabilization [43–45].

In conclusion, the data presented here provide the first ultrastructural documentation that oxidative stress alters the subcellular distribution of beclin-1 and suggest that the protein may be actively involved in the regulation of autophagy by participating in the mechanism of autophagosomes formation. We also demonstrate that autophagy is characteristic of the response of macrophages to oxidative insults and presumably, in this scenario, it may promote the selective clearance of dysfunctional proteins and organelles. The threshold between autophagy as prosurvival or prodeath process is difficult to establish and probably depends on the rate and extent of degradation of cellular components upon oxidant exposure. Since excess or prolonged autophagy has been proved to lead to autophagic cell death, the dramatic increase of autophagic vacuoles frequently observed in our series might represent an overstimulation of this process, an irreversible step indicative of a final cell demise. From our and similar studies, the current consensus is that autophagy possesses both adaptive and maladaptive features, depending on the degree of activation and nature and duration of the injury with the final conclusion that additional studies are necessary to better understand many mechanistic aspects of this pathway and its dual role in life and death.
